# Pregnancy outcomes in women affected by juvenile idiopathic arthritis (JIA)

**Published:** 2014-09-17

**Authors:** Maria Giannina Alpigiani, Pietro Salvati, Serena Callegari, Renata Lorini

**Affiliations:** 1 Pediatrics, Istituto G. Gaslini, Genova, Italy

## Introduction

JIA is the most frequent form of persistent arthritis in children that begins at or before 16 years old. While outcome of pregnant women with RA is well-known, at best of our knowledge there are a few scientific works about pregnancy in JIA patients [1,2].

## Objectives

Our aim is to describe pregnancy outcomes in a case series of six women affected by JIA.

## Methods

We report on six cases of women affected by JIA with a median age of 32,8; median age at onset of 6,1; median age at first delivery of 25,7. (table 1)

**Table 1:** 

	Age (y,m)	Age at onset	Type	Therapy before pregnancy	**Age at delivery** [pS1] **(y m)**	Sex of babies	Flare after delivery
**Patient 1** **LS**	29 7/12	12 y 2/12	poliarticular	None	18 11/12 24 7/12	♂♂	yes

**Patient 2** **GM**	38 7/12	8 y	poliarticular	none	27 3/12	♀	no

**Patient 3** **GA**	29 11/12	4 y	oligoarticular	none	25 9/12	♂	no

**Patient 4** **CE**	37 11/12	1 y 4/12	poliarticular	none	26 9/12	♀	yes

**Patient 5** **RL**	34 5/12	8 y 7/12	poliarticular	Cya, steroids	29 3/12	♀	yes

**Patient 6** **BA**	26 10/12	2 y 8/12	poliarticular	none	26 2/12	♂	yes

[pS1]

## Results

In all cases, pregnancy was associated with remission of disease activity, however a post partum flare appeared after 4 pregnancies ( pt 1-4-5-6) and in the first year post-partum. The seven babies were in good condition, without apparent malformation or symptoms of neonatal illness. Only 1 woman was treated during her pregnancy: the number 5 patient received oral cyclosporine for the first 5 months of pregnancy and oral low-dose corticosteroids for all pregnancy; she had an active disease before pregnancy and she had an important flare a few months after delivery.

As reported for pregnant patients affected by RA (Dolhain RJEM 2010), in our cases pregnancy was associated with a remission of disease in 6/6 patients and flare in post-partum period in 4/6 patients, probably depending on increased levels of serum alfa 2 glycoprotein and elevated levels of sex hormones that influence a shift in cytokine production from a Th1 to a Th2 profile. In fact, oestrogens inhibit T-cell function, progesterone stimulates Th2 effects and cortisol has a general immunosuppressive effect.

The number 5 patient was treated with cyclosporine and steroids. No congenital anomalies or increase of death rate were observed in infants exposed to cyclosporine antenatally. Besides low-dose steroids therapy (5-15 mg prednisone daily) does not increase low-birth-weight or small for gestation age infants.

## Conclusion

In conclusion, in JIA patients, a stable disease or remission should be reached before pregnancy and should be used safe immunosuppressive drugs to avoid a flare during pregnancy and in post-partum period.

## Disclosure of interest

None declared.
